# Splenic flexure colon cancer may represent a distinct prognostic subtype associated with elevated systemic inflammation and impaired nutritional status

**DOI:** 10.3389/fimmu.2026.1835428

**Published:** 2026-06-09

**Authors:** Zhi-Hua Yang, Yue Peng, Rong Shang, Xiao-Yan Tang, Yi-Wu Zhou, Xiao-Juan Li, Yu-Cui Liao

**Affiliations:** 1 Jiangxi Province Key Laboratory of Immunology and Inflammation, Jiangxi Provincial Clinical Research Center for Laboratory Medicine, Department of Clinical Laboratory, The Second Affiliated Hospital, Jiangxi Medical College, Nanchang University, Nanchang, Jiangxi, China; 2 School of Public Health, Jiangxi Medical College, Nanchang University, Nanchang, Jiangxi, China; 3 Department of Gastroenterology, The Central Hospital of Shangrao, Shangrao, Jiangxi, China; 4 Department of Hospital Infection Management, Kunming Children’s Hospital, Affiliated Children’s Hospital of Kunming Medical University, Kunming, Yunnan, China; 5 Biological Resource Center, Department of Academic Affairs, The Second Affiliated Hospital of Nanchang University, Nanchang, Jiangxi, China

**Keywords:** splenic flexure, inflammation, biomarker, Colorectal cancer, prognosis

## Abstract

**Background:**

Splenic flexure colon cancer (SFCC) is a rare gastrointestinal malignancy distinguished by its unique anatomical location and dual vascular supply. Nevertheless, its clinical behavior, prognostic outcomes, and underlying risk factors remain inadequately defined.

**Methods:**

A two-cohort study design enrolled 478 patients with stage II-III colon cancer. A colon cancer cohort (2012-2017) compared survival outcomes across tumor locations, while a separate SFCC-specific cohort (2012-2022) evaluated prognostic biomarkers in SFCC patients. A composite nutrition-inflammation integrating index (NII), derived from FPMLR and FPNLR, was developed and evaluated using Kaplan-Meier curves and Cox regression models.

**Results:**

Significantly worse recurrence-free survival (RFS) and overall survival (OS) were observed in patients with SFCC or ascending colon cancer (ACC) compared with those with transverse or descending colon cancers (TCC or DCC), in both colon cancer cohort and SFCC cohort. SFCC was characterized by higher TNM stage, lymph metastasis status, large tumor-size, poor differentiation, and inflammation-nutrition imbalance relative to TCC and DCC. Multivariate Cox regression analysis identified the NII as an independent prognostic factor for OS in SFCC (adjusted HR = 3.834, 95%CI=1.452-10.122). Importantly, NII remained significantly associated with unfavorable clinical outcomes in subgroups, including patients with normal level of CA19-9, stage III disease, and those who underwent adjuvant chemoradiotherapy.

**Conclusion:**

SFCC may represent a potentially distinct prognostic subtype of colon cancer that appears to be associated with relatively aggressive pathological features and a persistently elevated systemic inflammatory state. The NII may serve as an exploratory indicator of the chronic inflammation-nutrition imbalance in these patients, and it may offer modest added value for risk stratification and individualized management. Further large-scale, multicenter prospective studies are warranted to validate these findings.

## Introduction

Colorectal cancer (CRC) remains the second most common malignancy and the fourth leading cause of cancer-related mortality in China, despite substantial clinical advances in surgical techniques, systemic therapy, and molecular stratification strategies (1). Current clinical decision-making for CRC increasingly incorporates the disease with primary tumor location, which reflects well-recognized differences in embryologic origin, molecular profiles, tumor microenvironment, and response to systematic treatments (2–4). However, this simplified anatomic classification may obscure clinically relevant heterogeneity within each colonic subsite, particularly in splenic flexure colon cancer (SFCC).

The splenic flexure represents a distinct anatomical and clinical entity within the colon. It lies at the junction of the transverse colon and the descending colon and constitutes a vascular watershed zone. This unique vascular anatomy renders surgical resection technically demanding, frequently requiring extensive colonic mobilization and individualized operative strategies (5–7). It has historically been grouped with left-sided colon cancer in both clinical practice and research (8). Accumulating evidence suggests that SFCC exhibits molecular, microenvironmental, and prognostic features that are neither fully aligned with right- nor left-sided disease (9, 10), however, its clinicopathological uniqueness and independent prognostic relevance remain poorly characterized.

It has been well established that systematic inflammation exerts profound effects on genomic instability, tumor immune-inflammation microenvironment remodeling, and treatment resistance (11). Concurrently, cancer-related malnutrition is recognized to synergize with systemic inflammation, thereby impairing immune surveillance, treatment intolerance, and adverse survival outcomes in CRC (12). However, whether inflammation-nutrition differ along the longitudinal axis of the colon or whether such spatial heterogeneity contributes to site-specific tumor behavior remains largely unexplored. Moreover, data characterizing the inflammatory and nutritional landscape of SFCC are scarce.

In our previous study, we identified a non-linear distribution of chronic inflammatory burden along the colon, featuring a distinct secondary peak at the SFCC (13). Importantly, this inflammatory enrichment was accompanied by inferior clinical efficacy of adjuvant or systematic therapy and poorer survival outcomes (14). Further clinicopathologic evaluation revealed that these adverse prognosis could not be fully attributed to surgical complexity, postoperative complications, or disparities in adjuvant treatment, implicating intrinsic tumor biology, host systemic inflammation, and nutritional status might collectively drive prognosis. So, we speculate that SFCC may represent a biologically distinct subtype of colon cancer characterized by a heightened inflammatory-nutritional imbalance.

To comprehensively elucidate the characteristics of SFCC and the role of inflammation-nutrition balance in the disease, we established a colon cancer cohort (412 patients with colon cancer) and a SFCC (90 patients) cohort. Within the colon cancer cohort, we systematically compared survival outcomes, clinicopathological malignant feature, treatment, postoperative complications, and inflammation-nutrition balance between SFCC and other anatomically located colon cancer. We validated the survival differences between these groups and evaluated the prognostic value of 12 inflammation-nutrition ratios in the SFCC cohort. Finally, we developed and validated a novel nutrition-inflammation integrating index (NII) and demonstrated its clinical utility for prognostic risk stratification and personalized decision-making for clinic.

## Materials and methods

Colon cancer patients who underwent radical resection for ascending colon cancer (ACC), hepatic flexure colon cancer (HFCC), transverse colon cancer (TCC), SFCC, and descending colon cancer (DCC) were screened for eligibility at the Second Affiliated Hospital of Nanchang University and the Central Hospital of Shangrao (January 2012 and December 2017). Tumor location was determined based on surgical and pathological examination records. The splenic flexure was anatomically defined as the segment encompassing the distal transverse colon to the proximal descending colon. All included patients had histologically confirmed stage II-III colon cancer diagnosed for the first-time, with no prior neoadjuvant therapy or coexisting malignancy. Exclusion criteria comprised recent infection, autoimmune, chronic kidney disease, hematologic, cardiovascular, or cerebrovascular disease. Meanwhile, those with hereditary polyposis syndromes or inflammatory bowel disease were also excluded from the study. Ultimately, 412 eligible patients were included in the colon cancer cohort to compare survival outcomes and systemic chronic inflammation among colon cancer subgroups with different primary tumor locations. The significant survival differences would be validated between a separate SFCC cohort and other anatomically located colon cancers from the colon cancer cohort. Consequently, a separate SFCC cohort of 90 patients initially diagnosed with SFCC between January 2012 and December 2022 was enrolled to investigate the prognostic value of candidate systemic inflammatory biomarkers. All eligible patients have signed the informed consent forms upon hospital admission, explicitly authorizing the storage and future research use of residual clinical specimens (e.g., serum, plasma, EDTA blood, formalin-fixed paraffin-embedded tissue blocks) generated during standard care. The protocol of this study was approved by the Ethics Committee of the Hospital (O-MedResEthicsRev[2025] No. 97) and conducted in accordance with the Declaration of Helsinki.

We retrospectively collected the baseline and pathological characteristics of each eligible patient from the hospital electronic health records, including age, gender, comorbidities (diabetes and hypertension), status of weight loss, Eastern Cooperative Oncology Group (ECOG) score, primary tumor location, TNM stage, maximal tumor diameter, histologic subtype, tumor differentiation grade, presence of lymphovascular invasion, perineural invasion, and status of resection margins. Treatment-related variables, including surgical approach (open *vs.* minimally invasive), extent of lymphadenectomy, documented postoperative complications, and postoperative adjuvant chemoradiotherapy, were also systematically obtained from the hospital electronic medical records.

Preoperative peripheral blood samples were collected from the patient in a fasting state on the second morning of hospitalization (06:00–09:00). We detected laboratory parameters including complete blood counts (neutrophil, monocyte, lymphocyte, platelet), serum albumin (Alb), pre-albumin (pAlb), carcinoembryonic antigen (CEA), and carbohydrate antigen 19-9 (CA19-9), as well as plasma fibrinogen (Fib) concentration. The intra-assay and inter-assay coefficient of variation for all laboratory measurements were less than 10%. Nutritional status was primarily evaluated using Alb and pAlb, given their well-documented sensitivity to acute changes in both nutritional status and systemic inflammation. Prognostic nutrition index (PNI), neutrophil-to-lymphocyte ratio (NLR), platelet-to-lymphocyte ratio (PLR), and monocyte-lymphocyte ratio (MLR), Alb to Fib ratio (AFR), and Fib to pAlb ratio (FPR) were selected to assess systemic inflammatory status. To comprehensively capture the interplay among inflammation, immune response, coagulation activity, and nutritional status, we developed eight composite ratios such as three combining FPR with cellular ratios (FPNLR, FPPLR, FPMLR) and three combining AFR with cellular ratios (FANLR, FAPLR, and FAMLR). The detailed calculated formulas for all ratios were listed in **Supplementary Table 1**.

We conducted follow-up assessments every three months during the first two years and each six months during the third year. At each follow-up visit, patients underwent periodic clinical evaluations, laboratory testing, imaging studies, or colonoscopic examinations. Follow-up data were collected through review of medical records, telephone, and email. Recurrence-free survival (RFS) was the primary endpoint, and overall survival (OS) was the second endpoint. RFS was defined as the time from initial diagnosis to the occurrence of local recurrence or distant metastasis, whereas OS was defined as the time from initial diagnosis to death from any cause; patients without these events were censored at the date of their last follow-up.

In this study, continuous variables are presented as mean ± standard deviation or median with interquartile range, as appropriate, and are compared using Student’s t-test or the Mann-Whitney U test. Categorical variables are expressed as frequencies and percentages and compared using the Chi-square test or Fisher’s exact test. The optimal cut-off values of each inflammatory score were calculated by X-tile software (15), based on RFS and OS in overall patients with SFCC and ACC. Survival differences were estimated using the Kaplan–Meier method (log-rank test). Cox proportional hazards regression models were employed to identify independent prognostic factors. We firstly identified the significant biomarker using univariable Cox regression analysis, then the significantly characteristics were enrolled as confounding factors to adjust the hazard ratio (HR) and 95% confidence interval (CI) for the significant inflammatory biomarkers using backward, likelihood ratio test. The independent systemic inflammation-nutrition biomarkers were enrolled into a multivariable Cox regression model using backward, likelihood ratio test. Consequently, each coefficient of the significant biomarker was rigorously obtained to develop a new NII. To minimize potential overfitting and assess the robustness of variable selection, LASSO-Cox regression analysis was performed. The results were used to evaluate the stability of candidate predictors and to confirm consistency with the multivariable Cox model. Time-dependent receiver operating characteristic (ROC) curves were used to compare the predicted efficacy of these inflammation-based ratios. All statistical analyses were performed using SPSS Statistics version 26.0 software (IBM, Armonk, NY, USA.), R 4.5.2 (Institute for Statistics and Mathematics, Vienna, Austria) with packages of “survival” and “survminer”, and GraphPad Prism version 10.4.1 software (GraphPad Software Inc., San Diego, USA). A two-sided *p* < 0.05 was considered statistically significant.

## Results

### Patient baseline characteristics and the unique prognosis of SFCC

**Figure 1:**
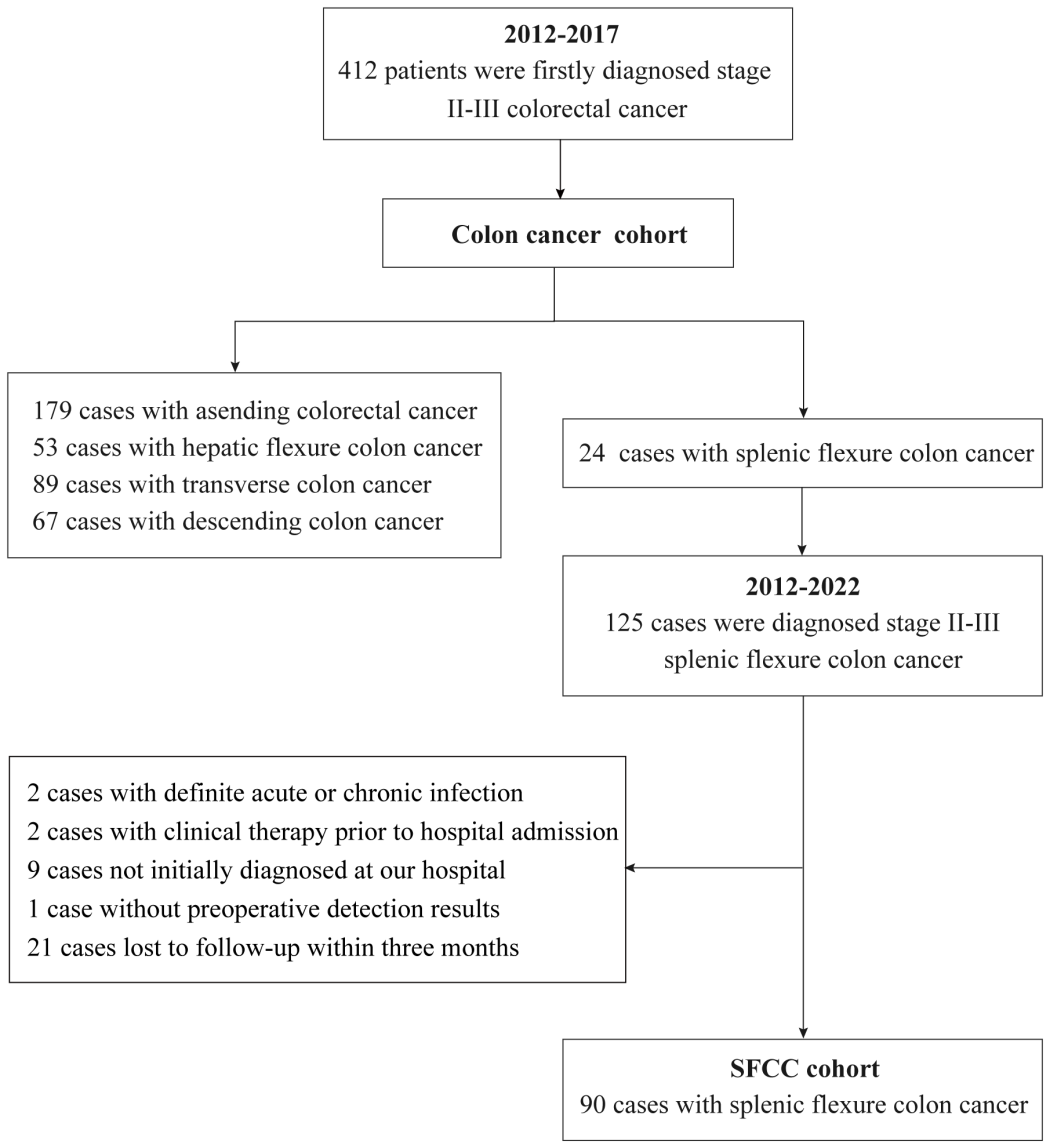
Screening and identifying flowchart of eligible patients in the present study.

A detailed patient selection flowchart is presented in **Figure 1**. As shown in **Figure 1**, a total of 179 ACC, 53 HFCC, 89 TCC, 24 SFCC, and 67 DCC patients who underwent curative resection were included in the colon cancer cohort according to the inclusion and exclusion criteria. Ninety SFCC patients were enrolled separately as the SFCC cohort. Baseline characteristics of the colon cancer cohort and SFCC cohort were summarized in **Table 1**; **Supplementary Table 2**, respectively. SFCC accounted for approximately 5.83% of the colon cancer cohort, and 35 (38.90%) and 20 (22.20%) of the patients were recurrent and dead during the follow-up period. Baseline demographic characteristics, including age and sex, were generally balanced across tumor locations. All enrolled patients had an ECOG performance status score of 0-2, with the proportions of scores 0, 1, and 2 being 4.22%, 94.37%, and 1.41%, respectively. The prevalence of major comorbidities, such as diabetes mellitus and cardiovascular or cerebrovascular disease, did not differ significantly between patients with SFCC and those with cancer in adjacent colonic segments. However, significant differences were observed in TNM stage, lymph node (LN) status, tumor size, and histologic differentiation between these groups. SFCC exhibited a significantly higher proportion of pathological aggressive features, including high stage III (*p* = 0.035), high LN status (*p* = 0.042), large tumor-size (*p* = 0.043) and poor differentiation (*p* = 0.033), compared with TCC or DCC (**Supplementary Figure 1**). Although serum levels of CEA and CA19–9 did not differ statistically across groups, the proportion of patients with elevated concentrations of both biomarkers was highest in the SFCC subgroup. The majority of patients received postoperative adjuvant chemoradiotherapy, and the proportion of patients undergoing the treatment did not differ significantly by tumor location. Despite the well-documented technical challenges associated with splenic flexure surgery, no significant difference in the overall postoperative complication rate was observed between SFCC and cancer arising from other colonic segments (**Table 1**).

**Table 1:** Comparison of clinical baseline characteristics according to tumor location in the colon cancer cohort.

Parameters	Ascending colon	Hepatic flexure	Transverse colon	Splenic flexure	Descending colon	*p-*value
	(N = 179)	(N = 53)	(N = 89)	(N = 24)	(N = 67)	
Sex (male)	100 (55.90%)	33 (62.30%)	55 (61.80%)	13 (54.20%)	42 (62.70%)	0.762
Age (≥ 60 years)	99 (55.30%)	35 (66.00%)	40 (44.90%)	13 (54.20%)	32 (47.80%)	0.132
Smoking (yes)	29 (16.20%)	8 (15.10%)	18 (20.20%)	3 (12.50%)	4 (6.00%)	0.165
Drinking (yes)	15 (8.40%)	6 (11.30%)	12 (13.50%)	2 (8.30%)	2 (3.00%)	0.188
Diabetes (yes)	14 (7.80%)	4 (7.50%)	4 (4.50%)	4 (16.70%)	5 (7.50%)	0.385
Hypertension (yes)	29 (16.20%)	11 (20.80%)	19 (21.30%)	7 (29.20%)	12 (17.90%)	0.557
TNM stage (III)	86 (48.00%)	17 (32.10%)	33 (37.10%)	15 (62.50%)	25 (37.30%)	**0.035**
LN status (N1-2)	86 (48.00%)	17 (32.10%)	33 (37.10%)	15 (62.50%)	26 (38.80%)	**0.042**
Tumor size (≥ 5 cm)	107 (60.50%)	37 (69.80%)	53 (59.60%)	11 (45.80%)	30 (44.80%)	**0.043**
Tumor type (MAC)	28 (15.70%)	11 (20.80%)	14 (15.90%)	4 (22.20%)	9 (13.60%)	0.805
Differentiation (poor)	25 (14.80%)	1 (2.00%)	11 (12.60%)	4 (23.50%)	5 (7.70%)	0.033*
Chemoradiotherapy (yes)	138 (77.10%)	40 (75.50%)	73 (82.00%)	22 (91.70%)	47 (70.10%)	0.195
CEA (≥ 5 ng/mL)	70 (40.70%)	15 (31.90%)	27 (31.00%)	18 (78.30%)	16 (24.60%)	0.144
CA19-9 (≥ 37U/mL)	46 (26.90%)	12 (25.50%)	20 (23.30%)	18 (78.30%)	12 (18.50%)	0.759
Alb (g/L)&	39.84 (35.86-42.30)	39.75 (36.94-43.11)	39.89 (37.08-41.96)	38.96 (35.63-41.88)	40.67 (37.72-42.70)	0.137^#^
preAlb (mg/L)&	173.31 (163.07-183.55)	187.50 (169.32-205.67)	183.61 (169.72-197.51)	165.53 (132.56-198.50)	188.11 (169.52-206.69)	0.356
Complications (yes)	36 (21.20%)	10 (19.60%)	9 (10.30%)	3 (15.80%)	11 (16.70%)	0.299
Gastrointestinal complication (yes)	23 (13.50%)	2 (3.90%)	7 (8.00%)	2 (10.50%)	3 (4.50%)	0.127
Infectious complication (yes)	13 (7.60%)	4 (7.80%)	5 (5.70%)	1 (7.60%)	5 (7.60%)	0.978
Other complications (yes)	13 (7.60%)	6 (11.80%)	3 (3.40%)	1 (7.60%)	5 (7.60%)	0.423

*compared with the Fisher’s exact test; ^#^compared with the rank-sum test; others compared with the chi-square test; ^&^: median (25% quartile to 75% quartile); TNM, Tumor-Node-Metastasis; LN, lymph node; MAC, mucinous adenocarcinoma; CEA, carcino embryonic antigen; CA19-9, carbohydrate antigen 19-9; Alb, albumin; preAlb, prealbumin.

The bold values indicate statistically significant results (p < 0.05).

**Figure 2:**
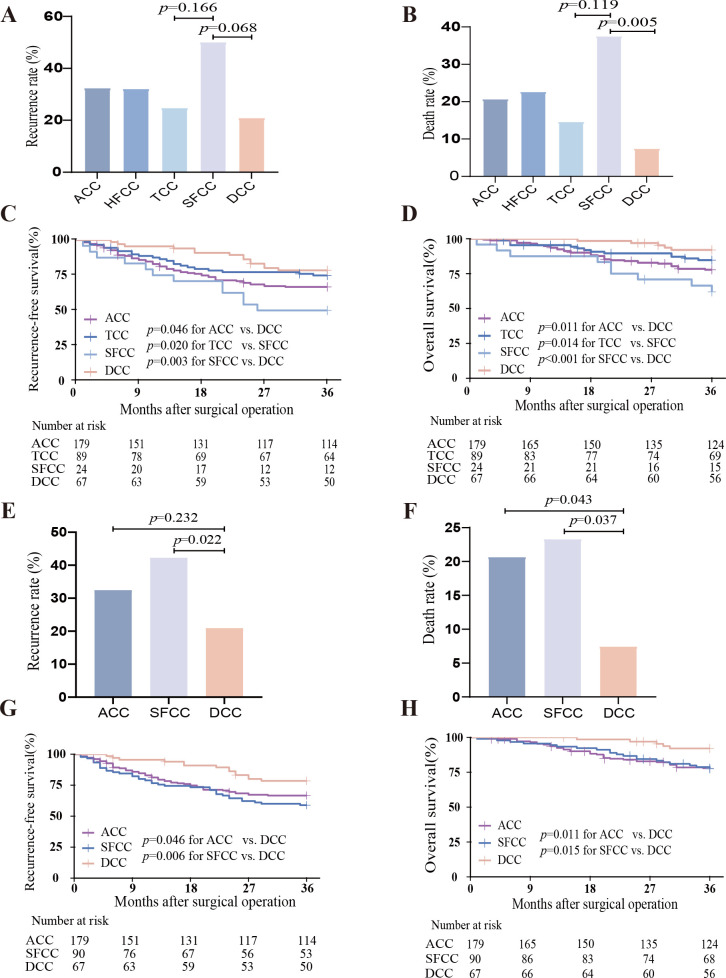
Comparison of prognostic outcomes according to primary tumor location in colon cancer cohort and SFCC cohort. Tumors are abbreviated as follows: ACC, Ascending Colon Cancer; HFCC, Hepatic Flexure Colon Cancer; TCC, Transverse Colon Cancer; SFCC, Splenic Flexure Colon Cancer; DCC, Descending Colon Cancer. **(A,B)** recurrence and death rate in the colon cancer cohort; **(C, D)** Kaplan-Meier curve analysis of survival in the colon cancer cohort; **(E, F)** recurrence and death rate in the SFCC cohort; **(G, H)** Kaplan-Meier curve analysis of survival in the SFCC cohort.

The highest recurrence (50.00%) and mortality rate (37.50%) were observed in the SFCC subgroup (**Figures 2A, B**), and the mortality difference was pronounced when comparing SFCC with DCC (37.50% *vs.* 7.50%, *p* = 0.005). Patients with SFCC exhibited significantly worse OS than those with TCC or DCC. A comparable trend was evident for RFS, with earlier recurrence and poorer long-term outcomes in the SFCC group. Notably, the survival outcomes of SFCC more closely resembled those of ACC than those of anatomically adjacent left-sided segments (**Figures 2C, D**).

Importantly, multivariable Cox proportional hazards regression analysis revealed that the postoperative risk of recurrence was higher in the SFCC group compared with the ACC, TCC, and DCC groups, with adjusted HRs of 1.627 (95% CI: 0.873-3.033; *p* = 0.125), 1.996 (95% CI: 0.985-4.046; *p* = 0.055), and 3.121 (95% CI: 1.284-7.587; *p* = 0.012), respectively. Similarly, the risk of death was also elevated in the SFCC group versus the ACC, TCC, and DCC groups, with adjusted HRs of 1.869 (95% CI: 0.901-3.874; *p* = 0.093), 2.834 (95% CI: 1.143-7.027; *p* = 0.025), and 8.735 (95% CI: 2.170-35.160; *p* = 0.002), respectively (**Table 2**). Furthermore, the survival curves of SFCC aligned more closely with ACC patients than with DCC.

**Table 2:** Survival comparison between SFCC and other anatomically located colon cancers in the colon cancer cohort and SFCC cohort.

Comparisons	Colon cancer cohort				SFCC cohort			
	Recurrence-free survival		Overall survival		Recurrence-free survival		Overall survival	
	*p*-value	HR (95%CI)	*p*-value	HR (95%CI)	*p*-value	HR (95%CI)	*p*-value	HR (95%CI)
SFCC vs. ACC	0.125	1.627 (0.873-3.033)	0.093	1.869 (0.901-3.874)	0.203	1.307 (0.865-1.975)	0.789	1.078 (0.621-1.873)
SFCC vs. TCC	0.055	1.996 (0.985-4.046)	**0.025**	2.834 (1.143-7.027)	**0.034**	1.771 (1.405-3.002)	0.232	1.532 (0.762-3.080)
SFCC vs. DCC	**0.012**	3.121 (1.284-7.587)	**0.002**	8.735 (2.170-35.160)	**0.008**	2.314 (1.251-4.282)	**0.017**	3.299 (1.236-8.803)

*p*-value, the value of the log-rank test. HR, hazard ratio; 95%CI, 95% confidence interval; HR (95%CI), multivariate Cox regression analysis adjusted for sex, age, smoking, drinking, hypertension, diabetes, and chemoradiotherapy.

The bold values indicate statistically significant results (p < 0.05).

When comparing survival outcomes in the SFCC cohort, patients with SFCC exhibited consistently higher recurrence and mortality rates than those with TCC or DCC in the colon cancer cohort (**Figures 2E–H**). Specifically, the HR for recurrence was 1.771 (95% CI: 1.405-3.002; *p* = 0.034) for SFCC versus TCC, and 2.314 (95% CI: 1.251-4.282; *p* = 0.008) for SFCC versus DCC. Similarly, the HR for mortality was 1.532 (95% CI: 0.762-3.080; *p* = 0.232) for SFCC versus TCC and 3.299 (95% CI: 1.236-8.803; *p* = 0.017) for SFCC versus DCC (**Table 2**).

### Inflammation-nutrition imbalance in SFCC

**Figure 3:**
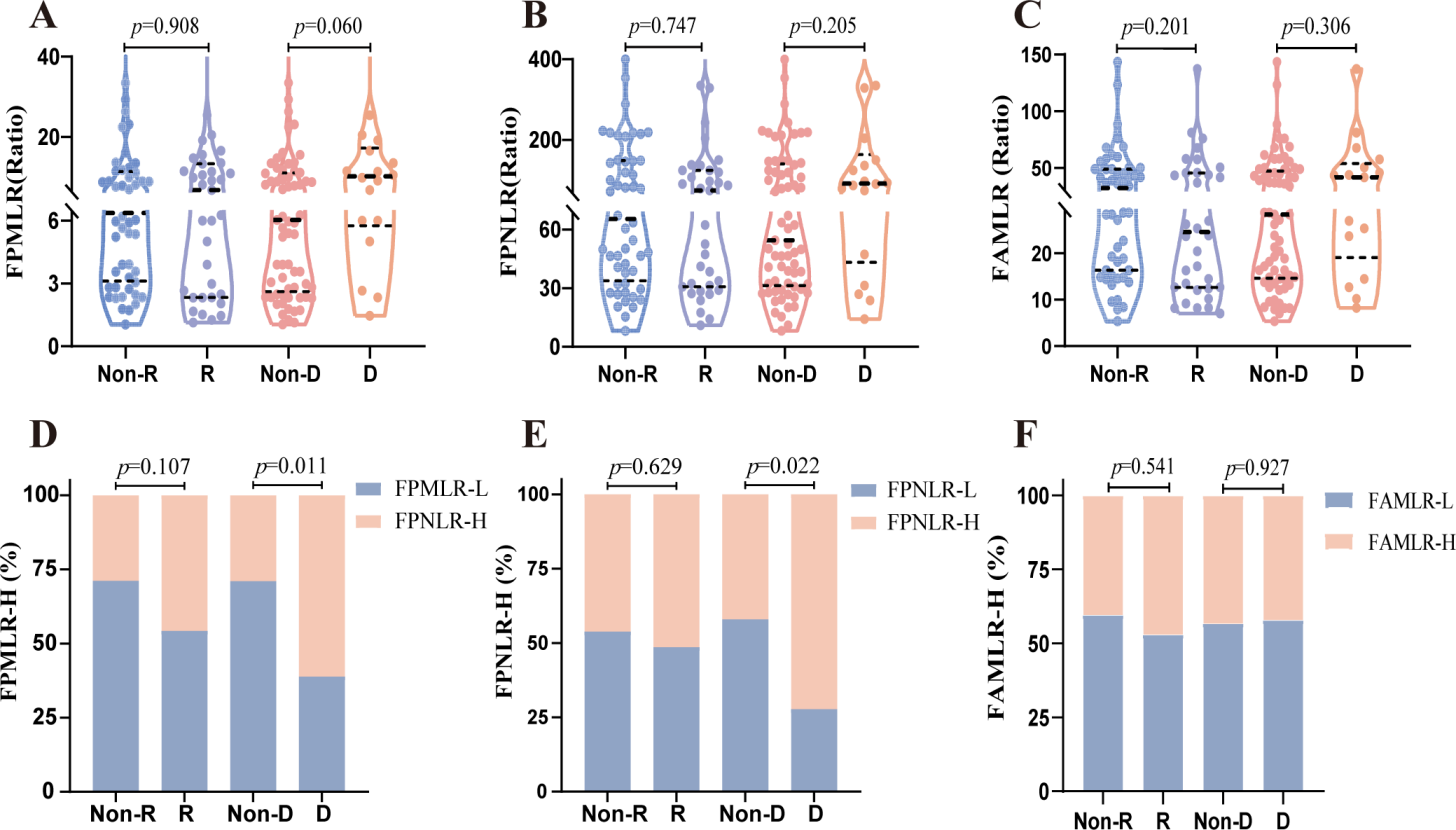
The prognostic value of inflammatory markers in the SFCC cohort. **(A-C)** FPMLR, FPNLR, and FAMLR as continuous variables between the recurrence (R) and non-recurrence groups (Non-R), as well as the death (D) and non-death groups (Non-D); **(D-F)** FPMLR, FPNLR, and FAMLR as categorical variables between the R and Non-R, as well as the D and Non-D.

To investigate whether the poor prognosis of SFCC stems from a nutrition-inflammation imbalance, we systematically compared systemic comprehensive inflammatory ratios across anatomical cancer locations in the SFCC cohort (**Supplementary Table 3**). Systemic inflammatory ratios demonstrated a non-linear distribution along the longitudinal axis of the colon. Patients with ACC and SFCC harbored the highest and second highest inflammatory burden, respectively, whereas TCC and DCC showed significantly lower chronic inflammation, particularly evident across multiple composite inflammation–nutrition ratios, including FPMLR (*p* = 0.015), FPNLR (*p* = 0.045), FPPLR (*p* = 0.028), FAMLR (*p* = 0.002), and FAPLR (*p* = 0.020) (**Supplementary Figures 2A–J**). The proportion of patients with elevated FPMLR (≥8.90) was highest in SFCC (36.80%), followed by ACC (33.70%), and lowest in DCC patients (13.10%). Similarly, elevated FPNLR (≥71.90) was observed in 48.30% of SFCC patients, versus 34.40% in ACC and 36.10% in DCC. Moreover, the proportion of patients with elevated inflammatory markers (FPMLR, FPNLR, and FAMLR) was higher among those who experienced SFCC recurrence or death (**Figure 3**). Nutritional biomarkers such as serum Alb and preAlb were significantly lowest in the SFCC subgroup of the colon cancer cohort. In contrast, TCC and DCC were associated with progressively attenuated systemic inflammation and a more favorable nutritional status. These results indicate that SFCC is not merely a transitional anatomical zone between right- and left-sided colon cancer, but rather a distinct regional phenotype characterized by pronounced systemic inflammation-nutritional dysregulation.

### Prognostic value of inflammatory biomarkers in SFCC

**Figure 4:**
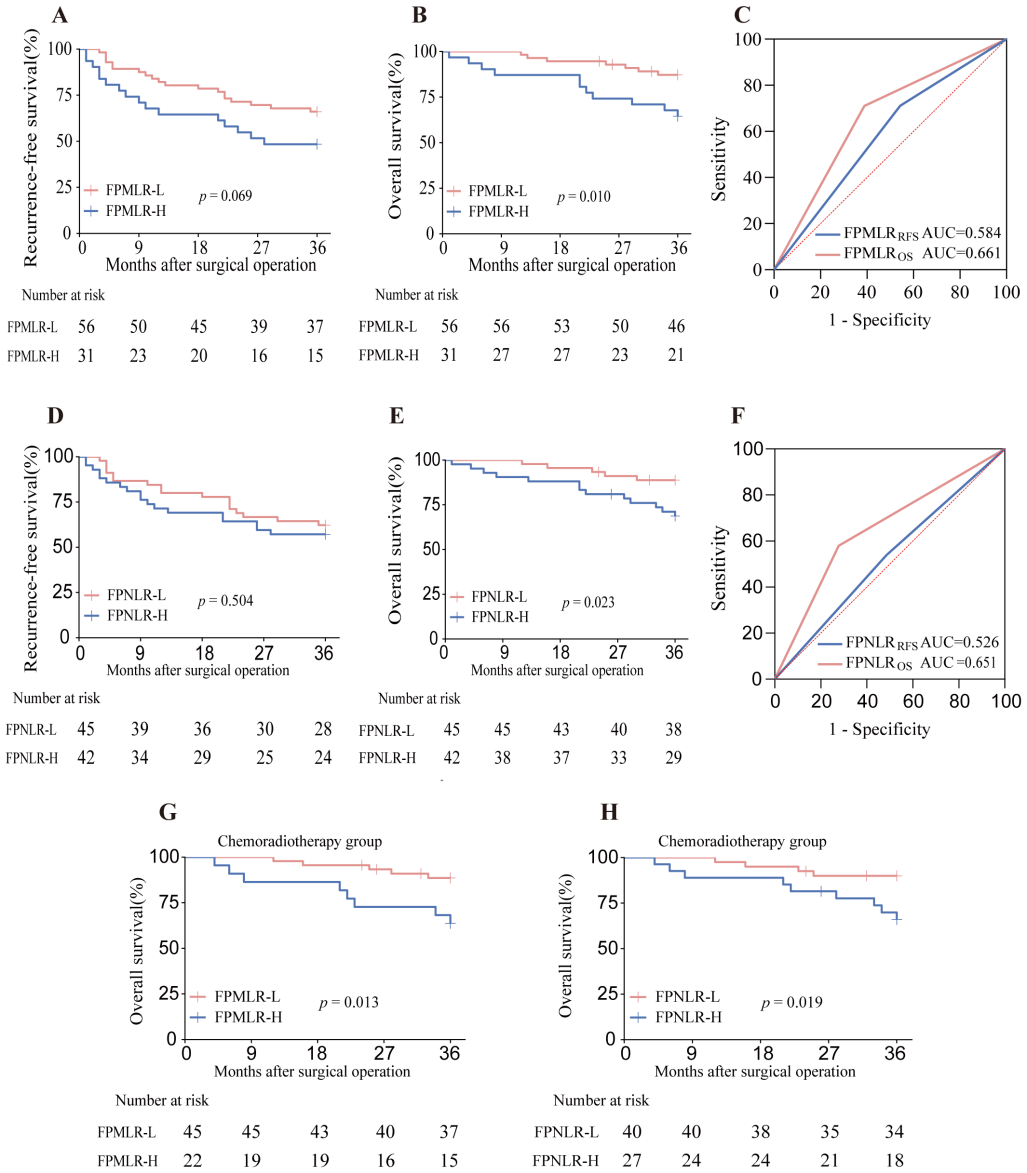
Prognostic evaluation and therapeutic efficacy prediction of inflammatory biomarkers in SFCC cohort. **(A-C)** Kaplan-Meier (K-M) curve of survival and the area under time-dependent ROC (AUROC) for predicting RFS and OS of FPMLR; **(D-F)** K-M curve of survival and AUROC of FPNLR; **(G-H)** K-M curve of chemoradiotherapy efficacy of FPMLR and FPNLR.

In the SFCC cohort, pathological characteristics including stage III and tumor differentiation were all significantly associated with both RFS and OS in univariate Cox regression (**Table 3**). Similarly, FPMLR, and FPNLR were each significantly associated with OS in univariate Cox regression. Multivariable Cox regression further identified hypertension (*p*_log-rank_=0.033, HR = 3.832, 95%CI=1.198-12.265), poor differentiation (*p*_log-rank_=0.002, HR = 5.325, 95%CI=1.818-15.597), elevated FPMLR (*p*_log-rank_=0.020, HR = 3.641, 95%CI=1.230-1.775), and elevated FPNLR (*p*_log-rank_=0.017, HR = 4.031, 95%CI=1.283-12.667) as independent factors for predicting OS of SFCC when adjusting for significant confounding factors in univariate Cox regression. Based on the number of recurrence or death patients and included confounding factors, the event-per-variable ratios were 17.5 for RFS and 6.7 for OS. Furthermore, the statistical powers of FPMLR and FPNLR for predicting OS were 0.790 and 0.880, which exceeded or approached the generally accepted level of 0.80. Time-dependent ROC analysis demonstrated that FPMLR achieved a superior predictive performance over other biomarkers for 36-month OS (AUC_36 month_=0.661) (**Figures 4A–F**). Furthermore, OS was significantly improved in low-FPMLR patients undergoing postoperative chemoradiotherapy compared to those with high FPMLR (**Figures 4G, H**).

**Table 3:** Univariate and multivariate analysis of clinical baseline characteristics and six novel inflammatory ratios in the SFCC cohort.

Parameters	Recurrence-free survival			Overall survival		
	Univariate		Multivariate	Univariate		Multivariate
	*p*-value*	HR (95%CI)	HR (95%CI)	*p*-value*	HR (95%CI)	HR (95%CI)
Sex (male)	0.116	0.771 (0.558-1.066)	–	**0.036**	0.624 (0.402-0.971)	0.498 (0.191–1.300)
Age (≥ 60 years)	0.501	1.249 (0.654-2.385)	–	0.060	2.506 (0.963-6.552)	–
Smoking (yes)	0.225	0.414 (0.099-1.721)	–	0.408	0.428 (0.057-3.198)	–
Drinking (yes)	0.340	0.380 (0.052-2.770)	–	0.865	0.840 (0.112-6.279)	–
Diabetes (yes)	0.585	0.672 (0.162-2.796)	–	0.611	1.461 (0.339-6.297)	–
Hypertension (yes)	0.588	0.797 (0.350-1.814)	–	0.644	1.270 (0.462-3.495)	–
Weight loss (yes)	0.546	1.322 (0.533-3.278)	–	0.347	0.376 (0.049-2.892)	–
TNM stage (III)	**<0.001**	4.092 (1.865-8.891)	3.647 (1.646-8.079)	**0.017**	3.782 (1.263-11.321)	2.763 (0.773-9.877)
Tumor size (≥ 5 cm)	0.184	0.643 (0.335-1.234)	–	0.655	0.819 (0.351-1.968)	–
Tumor type (MAC)	0.458	0.674 (0.237-1.912)	–	0.243	0.300 (0.040-2.264)	–
Differentiation (poor)	**0.019**	2.270 (1.144-4.502)	1.770 (0.882-3.552)	**0.001**	5.679 (2.095-15.395)	5.325 (1.818-15.597)
Chemoradiotherapy (yes)	0.351	1.516 (0.633-3.635)	–	0.431	0.666 (0.242-1.832)	–
CEA (≥ 5 ng/mL)	0.902	1.046 (0.514-2.126)	–	0.583	0.751 (0.270-2.086)	–
CA19-9 (≥ 37U/mL)	0.062	1.966 (0.967-3.995)	–	0.269	1.717 (0.659-4.473)	–
PLR (≥144.0)	0.564	1.225 (0.615-2.438)	–	0.540	1.349 (0.518-3.510)	–
FPMLR (≥8.9)	0.075	1.830 (0.941-3.561)	–	**0.015**	3.241 (1.256-8.364)	3.641 (1.230-10.775)
FPNLR (≥71.9)	0.508	1.251 (0.645-2.428)	–	**0.031**	3.111 (1.109-8.730)	4.031 (1.283-12.667)
FPPLR (≥61.7)	0.196	1.601 (0.784-3.270)	–	0.169	1.944 (0.753-5.014)	–
FAMLR (≥41.0)	0.502	1.260 (0.642-2.470)	–	0.975	1.015 (0.408-2.524)	–
FAPLR (≥16.5)	0.127	1.688 (0.961-3.310)	–	0.191	1.824 (0.741-4.491)	–

*p-value of log-rank test. HR, hazard ratio; 95%CI, 95% confidence interval; multivariate Cox regression analysis was adjusted by two confounding factors (TNM stage and differentiation) and three confounding factors (sex, TNM stage, and differentiation) for RFS and OS analyses, respectively.

The bold values indicate statistically significant results (p < 0.05).

### Development and validation of NII

**Figure 5:**
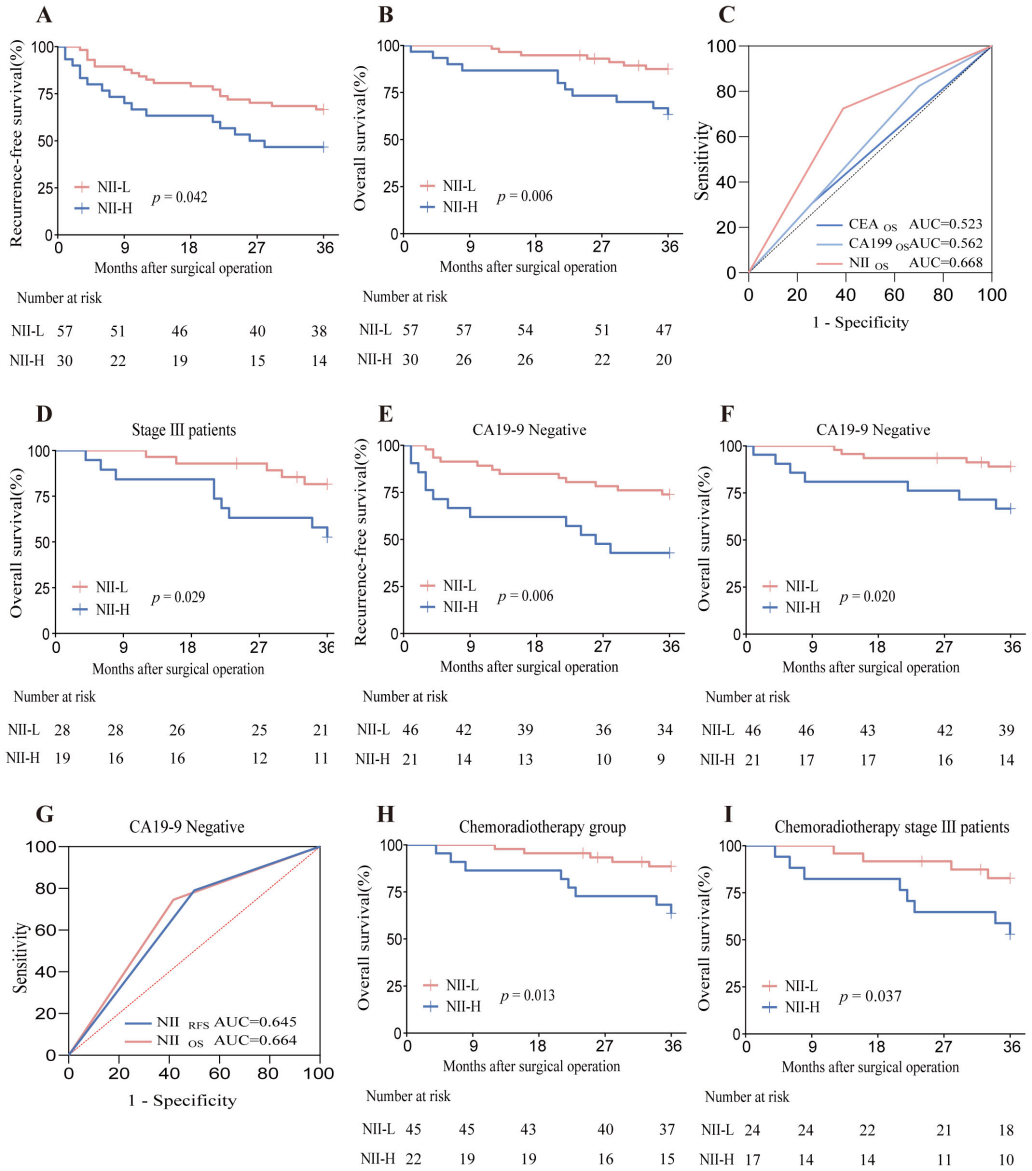
Prognostic and predictive value of NII in the SFCC cohort. (**A-C)** Kaplan-Meier (K-M) curve of survival and the area under time-dependent ROC (AUROC) for predicting OS in the SFCC cohort; **(D)** K-M curve of survival in patients with stage III; **(E-G)** K-M curve of survival and AUROC in CA19–9 negative group; **(H, I)** K-M curves of overall survival for patients undergoing chemoradiotherapy and stage III patients undergoing chemoradiotherapy.

To comprehensively evaluate the prognostic performance of inflammation- nutrition ratios in SFCC, we developed a novel NII derived from two independent systemic inflammation-nutrition biomarkers using multivariable Cox regression analysis, calculated as follows: NII = 0.035×FPMLR-0.001×FPNLR. Moreover, the variables selected by LASSO-Cox regression were highly consistent with those identified in the multivariable Cox regression model, suggesting good stability and robustness of the selected two prognostic factors. In the SFCC cohort, NII-H patients had significantly shorter RFS (*p*_log-rank_=0.042, **Figure 5A**) and OS (*p*_log-rank_=0.006, **Figure 5B**) than NII-Low patients. The NII achieved 36-month AUCs of 0.594 (RFS) and 0.668 (OS), significantly outperforming CEA (0.506 for RFS, 0.523 for OS) and CA19-9 (0.580 for RFS, 0.562 for OS), respectively (**Figure 5C**). It was a novel independent prognostic biomarker for both RFS (*p*_log-rank_=0.042, adjusted HR = 2.043, 95%CI=1.049-3.978) and OS (*p*_log-rank_=0.006, adjusted HR = 3.834, 95%CI=1.452-10.122) of SFCC, particularly for patients with the stage III disease (**Figure 5D**). More interestingly, NII remained an independent predictor of RFS and OS in multivariable models when modeled categorically across the SFCC cohort (**Supplementary Table 4**). Moreover, NII was significantly associated with worse RFS (*p*_log-rank_=0.006) and OS (*p*_log-rank_=0.020) (**Figures 5E, F**), achieving AUCs of 0.645 and 0.664 for 36-month RFS and OS, respectively, in the CA19-9-negative subgroup (**Figure 5G**). Similarly, among patients who received adjuvant chemoradiotherapy, NII-H predicted inferior OS (*p*_log-rank_=0.013, **Figure 5H**), with heightened prognostic significance in stage III cases (*p*_log-rank_=0.037, **Figure 5I**).

## Discussion

Although CRC remains one of the most prevalent and lethal malignancies worldwide, SFCC accounts for approximately 2%-8% of all CRC cases (16, 17). The clinical characteristics, prognostic outcomes, and underlying inflammation-nutrition imbalance in SFCC remain poorly characterized. In this study, we systematically described the clinicopathological features, systemic inflammatory–nutritional status, and survival outcomes of SFCC using both a general colon cancer cohort and an independent SFCC cohort. Our findings suggest that SFCC may be associated with less favorable oncologic outcomes compared with tumors arising from anatomically adjacent colonic segments, even after accounting for similar stage distribution, surgical approaches, and postoperative adjuvant therapy. In addition, SFCC appeared to be accompanied by relatively more aggressive histological features, a higher systemic inflammatory burden, and a concurrent impairment in nutritional status.

The traditional dichotomization of colon cancer into right- and left-sided disease has been widely adopted in clinical practice and translational research (18, 19). However, accumulating evidence suggests that this binary framework oversimplifies the biological heterogeneity of CRC, particularly for cancer arising at anatomically transitional sites such as the sigmoid flexure. In a large multicenter observational study of 17,641 patients, Benedix et al. reported that proximal colon cancer conferred significantly worse prognosis than distal disease; however, this survival disparity was markedly attenuated after multivariable adjustment for TNM stage and splenic flexure was considered as left-colon (9). Yamauchi et al. demonstrated that key molecular features, including CpG island methylator phenotype (CIMP), microsatellite instability (MSI), and BRAF mutations, exhibit a continuous, linear gradient from the rectum to the ascending colon, with no sharp inflection point at the splenic flexure (20). This “continuum model” implies that SFCC may harbor an intermediate molecular phenotype bridging right- and left-sided colon cancers. Consistent with these observations, our analysis showed that TCC and DCC had the lowest recurrence and mortality rates, whereas SFCC showed higher recurrence and death rates relative to both TCC and DCC. Patients with SFCC also tended to demonstrate inferior RFS and OS across both colon cancer cohort and SFCC cohort, with Kaplan-Meier survival curves showing a pattern more similar to that of ACC than DCC. These findings suggest that the prognostic behavior of SFCC may be more similar to that of right-sided CRC, which may challenge the conventional assignment of SFCC to the left-sided category.

Accumulated evidence indicates tumor biological aggressiveness, preoperative comorbidity burden, nutritional status, chronic systemic inflammation, surgical resection quality, postoperative complications, and adjuvant treatment strategies collectively influence survival outcomes in CRC (21). In the colon cancer cohort, we included patients with ACC, TCC, SFCC, and DCC, all of whom underwent radical resection. Preoperative comorbidity, postoperative complication rate, and the use of adjuvant chemoradiotherapy were broadly comparable across the anatomical subgroups. These findings suggest that differences in baseline clinical status, surgical management, or postoperative care are unlikely to fully explain the relatively poorer survival observed in SFCC. Notably, SFCC appeared to be associated with a higher proportion of adverse pathological features, including stage III, high LN status, large cancer-size, and poorer histological differentiation compared with TCC or DCC, which may indicate a more aggressive malignant phenotype. This observation is in line with previous reports by Cao et al. (22). The enrichment of these high-risk pathological characteristics in SFCC may partly contribute to its less favorable clinical outcomes and suggests potential biological differences beyond anatomical location alone. Preoperative nutritional assessment and postoperative nutritional support are well-established determinants of recovery and survival in CRC (23). In accordance with this, our analysis suggested a non-linear distribution of nutritional biomarkers along the colonic axis, with lower serum Alb and pAlb levels observed in SFCC, which may reflect a higher degree of nutritional impairment and could be associated with poorer prognosis.

Systematic chronic inflammation is widely recognized as a hallmark of CRC (24). Our prior studies showed that established inflammatory biomarkers including NLR, PLR, and LMR, and nutrition-inflammation combined ratios FPR and AFR, are independently associated with CRC prognosis (25). In this study, we observed a non-linear distribution of chronic inflammatory burden along the colonic axis, with relatively higher levels detected in right-sided colon cancer and SFCC, especially for biomarkers such as FPMLR, FPNLR, FPPLR, FAMLR, and FAPLR. Despite its conventional anatomical classification as a left-sided cancer, SFCC exhibited inflammatory, pathological, and prognostic features that closely parallel those of right-sided disease. The relatively elevated inflammatory phenotype may be partly related to its unique vascular supply and anatomical characteristics of the splenic flexure, although the underlying mechanisms remain speculative and require further investigation. Specifically, the splenic flexure lies at the watershed zone between the superior and inferior mesenteric artery territories, rendering it vulnerable to relative hypoperfusion, which may potentially trigger ischemia-reperfusion injury and sustain low-grade chronic inflammation (26). Furthermore, its anatomic proximity to the spleen enables locally advanced SFCC to directly invade the splenic parenchyma or form inflammatory adhesions via the splenocolic ligament, occasionally culminating in splenic abscess formation (27). As the largest peripheral immune organ, the spleen undergoes microenvironmental remodeling in response to cancer-associated stimuli, promoting expansion of myeloid-derived suppressor cells, thereby exacerbating systemic immunosuppression and chronic inflammation (28).

FPMLR, FPNLR, and NII, which integrate systemic inflammatory activity, immune cell dynamics, coagulation-related components, and nutritional status into a single composite index, may capture multiple aspects of host-tumor interactions. We found that both FPMLR and FPNLR were significantly associated with survival of SFCC, and appeared to retain independent prognostic value within this anatomical subgroup. The NII, exhibited modest prognostic performance for both RFS and OS in SFCC patients, with AUC values numerically higher than those of CEA and CA 19-9, as well as those previously reported for the Glasgow prognostic score (GPS) and modified GPS in radically operated patients with stage II-III CRC (29, 30). Improved predictive performance of the NII was observed in the CA19-9-negative and stage III subgroups. Importantly, among stage III patients, those with a low NII appeared to derive greater absolute benefit from adjuvant chemoradiotherapy, showing more favorable survival outcomes compared with those with high-NII. The statistical powers were 0.901 and 0.770 in the CA19-9-negative and stage III subgroups, respectively, which were close to or above the conventional threshold of 0.80. Collectively, these results suggest that the poorer prognosis observed in SFCC may be partly associated with intrinsic tumor biology and a persistent systemic inflammation-nutrition imbalance, rather than anatomical location alone. SFCC may therefore represent a clinically and biologically distinct subgroup. However, this interpretation remains tentative and requires further validation in large, independent, prospective cohorts.

In this study, our findings suggest SFCC may represent a potentially high-risk anatomical-biological subgroup, which may warrant further consideration in risk stratification and postoperative surveillance strategies. The NII may serve as a low-cost and easily accessible prognostic factor for identifying SFCC patients who are potentially at higher risk of recurrence and cancer-specific mortality. Integration of NII into postoperative clinical decision-making may have the potential to support more individualized management in the future. However, several limitations should be acknowledged. First, the retrospective design, single-center setting, and lack of an independent external validation cohort for FPMLR, FPPLR, and NII may limit the generalizability of our findings. Therefore, all prognostic performance estimates should be considered preliminary. In addition, the reported AUC values for the NII showed modest discriminative ability. Therefore, these results should be interpreted as preliminary and require validation in prospective, multicenter studies with large sample sizes prior to clinical application. Second, despite our best efforts to enroll patients with diagnosed SFCC, the small sample size of SFCC patients in our study remained relatively limited, and the events-per-variable ratio was below the recommended threshold in certain analyses. This may have reduced statistical power and increased the potential risk of model overfitting, particularly in the stage III and CA19-9-negative subgroups. Therefore, our primary findings should be validated in studies with larger sample sizes. Third, due to insufficient serum samples for CRP detection and an oversight in study design, we were unable to obtain reliable measurements of serum C-reactive protein, patient height and weight, or molecular and genomic profiling data (*KRAS*/*BRAF* mutation status, microsatellite instability, and consensus molecular subtypes). Consequently, we could not calculate GPS or modified GPS, nor could we determine BMI. Moreover, the absence of molecular and genomic profiling precluded integration of inflammatory–nutritional biomarkers with established CRC molecular subtypes. Future studies should prioritize prospective NII validation and mechanistic exploration of inflammation–nutrition crosstalk at the splenic flexure using spatial transcriptomics, immune microenvironment mapping, and longitudinal biomarker assessment. These efforts will advance the anatomical–biological subclassification of colon cancer and facilitate precision interventions targeting the unique host–cancer interface in SFCC.

In conclusion, SFCC may represent a distinct clinical–biological entity, potentially differing from the conventional right–left anatomical classification in terms of inflammatory and nutritional characteristics. The NII may be considered an exploratory indicator of site-specific chronic inflammation and nutritional imbalance, and it could provide modest support for risk stratification and personalized management in SFCC. However, these findings shall be interpreted with caution, as they are preliminary and based on a limited sample. Independent validation in large-scale, prospective, multicenter studies is essential before any clinical application can be considered.

## Data Availability

The raw data supporting the conclusions of this article will be made available by the authors, without undue reservation.

## Supplementary material

Supplementary Figure 1 Comparison of features across different tumor locations in the colon cancer cohort. **(A-D)** different locations and TNM III stage rate, high lymph node (LN) status rate, larger tumor-size rate, and poor differentiation rate.

Supplementary Figure 2 Comparison of inflammatory markers and clinical features across different tumor locations in the SFCC cohort. **(A-E)** FPMLR, FPNLR, FPPLR, FAMLR, and FAPLR as continuous variables; **(F-J)** FPMLR, FPNLR, FPPLR, FAMLR, and FAPLR as categorical variables.
