# Supplementary material for: Splenic flexure colon cancer may represent a distinct prognostic subtype associated with elevated systemic inflammation and impaired nutritional status
Source: Front Immunol. 2026 Jun 9;17:1835428. doi: 10.3389/fimmu.2026.1835428 (PMC13286845; doi:10.3389/fimmu.2026.1835428)

Supplemental Table 1. The definitions and cut-off values of twelve inflammatory ratios in present study.

Biomarkers	Calculation formulas	Cut-off value
PNI	Albumin + 5×Lymphocyte	46.0
FPR	Fibrinogen/Prealbumin×1000	26.8
AFR	Albumin/Fibrinogen	11.1
MLR	Monocyte/Lymphocyte	0.3
NLR	Neutrophil/Lymphocyte	3.7
PLR	Platelets/Lymphocyte	144.0
FPMLR	(Fibrinogen/Prealbumin)×(Monocyte/Lymphocyte)	8.9
FPNLR	(Fibrinogen/Prealbumin)×(Neutrophil/Lymphocyte)	71.9
FPPLR	(Fibrinogen/Prealbumin)×(Platelets/Lymphocyte)/100	61.7
FAMLR	(Monocyte/Lymphocyte)/(Albumin/Fibrinogen)×1000	36.2
FANLR	(Neutrophil/Lymphocyte)/(Albumin/Fibrinogen)×100	30.2
FAPLR	(Platelets/Lymphocyte)/(Albumin/Fibrinogen)	19.7

Note: the cut-off values of the ratios are calculated using X-tile software according to progression-free survival and overall survival in patients with splenic flexure colon cancer and ascending colon cancer.

Supplemental Table 2. The clinicopathological characteristics of the SFCC cohort.

Parameters	SFCC cohort (N=90)
Sex (male)	33(36.70%)
Age (≥ 60 years)	46(51.10%)
Smoking (yes)	9(10.00%)
Drinking (yes)	5(5.60%)
Diabetes (yes)	7(7.80%)
Hypertension (yes)	20(22.20%)
Weight loss (yes)	13(18.30%)
TNM stage (III)	49(54.40%)
LN status (N1-2)	49(54.40%)
Tumor size (≥ 5 cm)	48(53.30%)
Tumor type (MAC)	13(15.50%)
Differentiation (poor)	13(15.50%)
ECOG score (0-2)	90(100.00%)
Chemoradiotherapy (yes)	70(77.80%)
CEA (≥ 5 ng/mL)	26(29.90%)
CA19-9 (≥ 37U/mL)	18(20.50%)
Alb (g/L)*	39.76(38.80-40.72)
preAlb (mg/L)*	196.62(155.27-221.67)
PNI(≤46.0)	38(42.20%)

Abbreviation: TNM, Tumor-Node-Metastasis; LN, lymph node; MAC, mucinous adenocarcinoma; ECOG score, Eastern Cooperative Oncology Group score; CEA, carcino embryonic antigen; CA 19-9, carbohydrate antigen 19-9; Alb, albumin; preAlb, prealbumin; PNI, prognostic nutritional index. *: median (25% quartile -75% quartile).

Supplemental Table 3. Comparison of twelve inflammatory biomarkers according to tumor location in the colon cancer cohort.

Parameters	Ascending (N=179)	Transverse (N=89)	Splenic flexure (N=90)	Descending (N=67)	*p*-value
PNI (≤46.0)	85(45.90%)	40(21.60%)	38(20.50%)	22(11.90%)	0.222
FPR (≥26.8)	51(31.30%)	15(18.30%)	23(26.40%)	16(26.20%)	0.193
AFR (≥11.1)	93(53.10%)	53(60.20%)	43(50.00%)	42(64.60%)	0.219
MLR (≥0.3)	74(41.30%)	38(42.70%)	45(50.00%)	20(29.90%)	0.091
NLR (≥3.7)	59(33.30%)	29(32.60%)	36(40.00%)	17(25.40%)	0.290
PLR (≥144.0)	134(74.90%)	66(74.20%)	58(64.40%)	39(58.80%)	**0.037**
FPMLR (≥8.9)	50(33.70%)	22(26.80%)	32(36.80%)	8(13.10%)	**0.015**
FPNLR (≥71.9)	56(34.40%)	23(28.00%)	42(48.30%)	22(36.10%)	**0.045**
FPPLR (≥61.7)	50(30.70%)	14(17.10%)	23(26.40%)	9(14.80%)	**0.028**
FAMLR (≥36.2)	50(28.60%)	21(23.90%)	37(43.00%)	10(15.40%)	**0.002**
FANLR (≥30.2)	67(38.30%)	31(35.20%)	38(44.20%)	21(32.30%)	0.460
FAPLR (≥19.7)	76(43.40%)	34(38.60%)	35(40.70%)	14(21.50%)	**0.020**

Abbreviation: PNI, prognostic nutritional index; FPR, fibrinogen-prealbumin ratio; AFR, albumin-fibrinogen ratio; MLR, monocyte-lymphocyte ratio; NLR, neutrophil-lymphocyte ratio; PLR, platelets-lymphocyte ratio; FPMLR, FPR×MLR; FPNLR, FPR×NLR; FPPLR, FPR×PLR; FAMLR, FAR×MLR; FANLR, FAR×NLR; FAPLR, FAR×PLR.

Supplementary Table 4. Prognostic roles of FPMLR, FPNLR, and NII in SFCC cohort.

Parameters	Recurrence-Free Survival			Overall Survival		
	p-value*	Model a	Model b	*p*-value*	Model a	Model b
FPMLR (Per SD)	0.630	1.007(0.979-1.037)	1.028(0.992-1.064)	0.087	1.022(0.997-1.048)	1.037(1.002-1.072)
FPMLR (Median)	0.839	1.071(0.552-2.079)	1.128(0.542-2.348)	0.140	2.092(0.785-5.574)	3.068(0.864-10.897)
FPNLR (Per SD)	0.987	1.000(0.999-1.001)	1.001(0.999-1.002)	0.392	1.001(0.999-1.002)	1.001(1.000-1.003)
FPNLR (Median)	0.750	1.114(0.574-2.161)	1.000(0.488-2.050)	**0.050**	2.803(0.999-7.864)	1.904(0.616-5.338)
NII (Per SD)	0.476	1.532(0.475-4.946)	2.495(0.468-13.304)	0.069	2.883(0.919-9.046)	7.303(1.454-36.682)
NII cut-off (Median)	0.319	1.406(0.720-2.746)	1.137(0.518-2.496)	**0.046**	2.857(1.108-8.016)	4.114(1.001-16.991)
NII cut-off (≥0.23)[#]	**0.048**	1.960(1.007-3.814)	2.043(1.049-3.978)	**0.011**	3.441(1.333-8.880)	3.834(1.452-10.122)

Abbreviation: FPMLR, FPR×MLR; FPNLR, FPR×NLR; NII, nutrition–inflammation integrating index; SD: standard deviation. *: *p*-value of Kaplan-Meier curve; [#]: NII cut-off (≥0.23) was calculated by X-tile software in the SFCC cohort; Model a: crude hazard ratio and 95% confidence-interval; Model b: multivariate Cox regression analysis (adjusted hazard ratio and 95% confidence interval) adjusted by sex, age, smoking, drinking, diabetes, hypertension, TNM stage, tumor size, tumor type, differentiation, and chemoradiotherapy.

Supplementary Figure 1

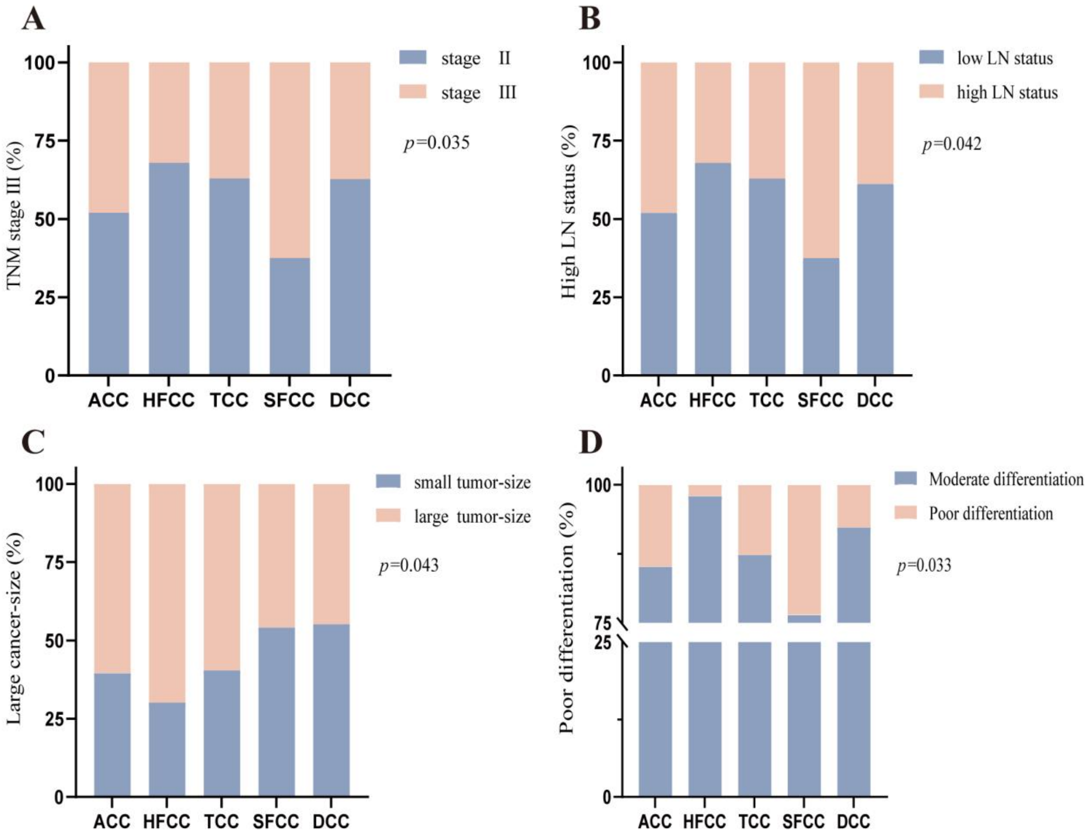

Supplementary Figure 2

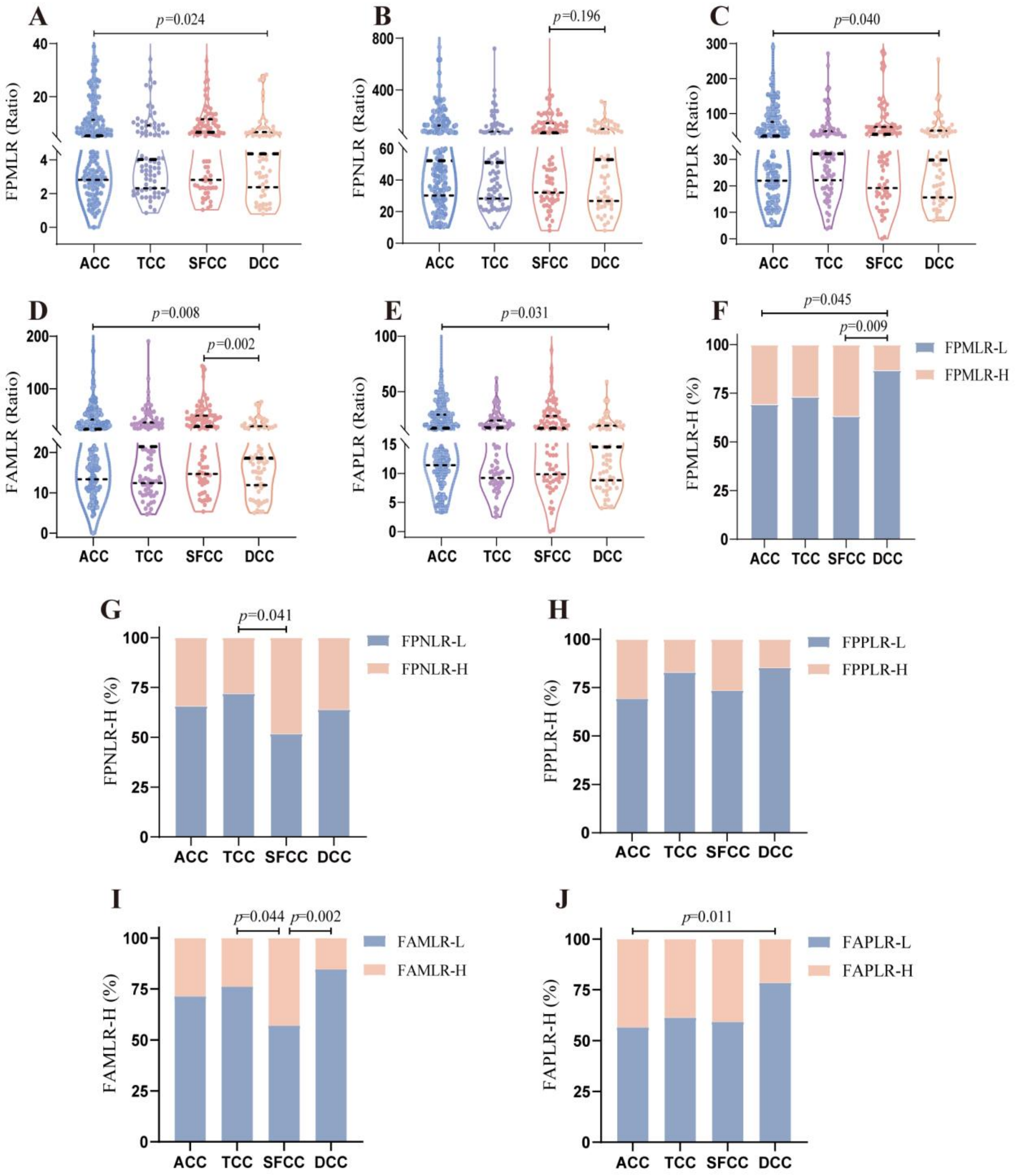

Supplement: Supplementary Figure 1 — Comparison of features across different tumor locations in the colon cancer cohort. (A-D) different locations and TNM III stage rate, high lymph node (LN) status rate, larger tumor-size rate, and poor differentiation rate. [file DataSheet1.pdf]
